# Editorial: Generative artificial intelligence and the ecology of human development

**DOI:** 10.1111/jcpp.13860

**Published:** 2023-09

**Authors:** Carlo Schuengel, Alastair van Heerden

**Affiliations:** 1 Educational and Family Studies, Vrije Universiteit Amsterdam, Amsterdam, The Netherlands.; 2 Amsterdam Public Health research institute, Amsterdam UMC and Vrije Universiteit Amsterdam, Amsterdam, The Netherlands.; 3 Center for Community Based Research, Human Sciences Research Council, Pietermaritzburg, South Africa; 4 SAMRC/WITS Developmental Pathways for Health Research Unit, Department of Paediatrics, School of Clinical Medicine, Faculty of Health Sciences, University of the Witwatersrand, Johannesburg, Gauteng, South Africa

## Abstract

Commercial applications of artificial intelligence (AI) in the form of Large Language Models (LLMs) and Generative AI have taken centre stage in the media sphere, business, public policy, and education. The ramifications for the field of child psychology and psychiatry are being debated and veer between LLMs as potential models for development and applications of generative AI becoming environmental factors for human development. This Editorial briefly discusses developmental research on generative AI and the potential impact generative AI on the hybrid social world in which young people grow up. We end by considering whether the implications justify increasing attention in our field.

There is no escaping the news about Large Generative AI Models (LGAIMs). Commercial applications of artificial intelligence in the form of Large Language Models (LLMs) and Generative AI (e.g., chatGPT; Dall-E) have taken centre stage in the media sphere, business, public policy, and education, quickly gaining traction outside the original scholarly field of computer science. Leading developmental researchers such as Michael Frank (2023) and Alison Gopnik (2023) have written on prominent academic platforms about how the science of child development may contribute to the understanding and even the education of LGAIMs. Furthermore, with the hybridization of online and offline social interactions, applications of generative AI are likely to rapidly become part of young people’s private sphere – transitioning us from the era defined by apps optimized to gain people’s attention to a new era that will focus on apps’ ability to form direct relationships with their users This is all unfolding at exponential rates, so much so that scholars, opinion makers, and policy makers are sounding the alarm and debating a moratorium to allow research, reflection, and regulation (Clarke, 2023).

The widespread introduction of generative AI is taking place at the heels of the COVID-19 pandemic, which was treated as a global emergency by the World Health Organization, leading to drastic public health measures and a redirection of scientific research programs on a massive scale (Ioannides et al., 2022). Families, practitioners, and policy-makers faced many urgent questions about this novel threat, and scientists responded beyond the obvious fields of immunology, infection prevention, and public health, aided by the quick reallocation of research funding and expedited translation into practice. These events have led to scientific breakthroughs in some fields (e.g., mRNA; infection control), while the impact in other fields remains to be seen.

Now again, the proliferation of LGAIMs and the prospect of artificial general intelligence generate many new questions and possibilities for breakthroughs, spurring governments to announce large investments in research and development and computing infrastructure. Once again, we may ask whether interests of youth and families are best served by trying not to become too distracted by this shiny new toy or whether in the public interest, we should engage early and comprehensively with new powerful tools.

## Developmental psychological research on generative AI

Developmental research conducted on LGAIMs has captured the imagination of many, with preprints of articles being widely circulated and debated. For example, Kosinski posted in February 2023 a preprint article reporting on a series of experiments suggesting that LGAIMs perform better on false beliefs tasks if these are trained on more language data (i.e., successive versions of GPT-3 and GPT-4), which he interpreted as demonstrating how Theory of Mind is an ability that can emerge from learning to put one word after the other. Another study quickly followed that showed that performance of LGAIMs on false belief tasks was not robust against variations in vignettes and prompts, supporting a more sceptic stance towards the emergence of higher cognitive functions in these models (Ullman, 2023). The issue is far from settled, however, and researchers may have started to consider how LLMs perform on other psychological tests and assessments and to ponder the implications of their initial observations of LGAIMs’ performance.

The current issue includes fascinating empirical work on aggressive behaviour in response to simulated rejection (Quarmley et al., 2023) and on parents’ ability to see the world from the point of view of their child with autism (Oppenheim et al., 2023). Both studies relate to the models that youth and adults construct of their social world and their place in it and both studies try to grapple with the problem that those models are opaque to social partners and can only be indirectly studied by researchers. The allure of research on LGAIMs is that, in principle, the models that underly complex social-cognitive abilities may be directly described and probed (Frank, 2023). However, such work may only start to become relevant for students of human behaviour and development outside linguistics, such as the readers of this journal, if LGAIMs can be demonstrated to show functional competencies (pairing linguistic knowledge with formal reasoning, world knowledge, situational modelling, and communicative intent) over merely formal competence (as a product of linguistic knowledge only; Mahowald et al., 2023). However, it is likely that someone somewhere is working to overcome the considerable challenges in combining technologies so that LGAIMs attain functional competencies.

Additional problems may need to be tackled as well, if generative AI is to catalyse our science in ways akin to the automation of gene sequencing and the arrival of affordable neuroimaging apparatus. For one, science has learned the hard way that reproducibility and replicability are key aspects of scientific rigour. This requires that either the commercial operators of LGAIMs implement and facilitate open science practices, such as persistent identifiers to the versions of their models and continued access, or that research and investment should focus on open-source LLMs (Spirling, 2023). Another set of problems relates to novel regulatory, legal, and ethical dilemmas that need to be resolved across the research value chain from institutes, funders, to publication outlets.

## Generative AI in a hybrid social world

It may provide some comfort to parents, professionals, historians, and policy makers that developmental and cognitive research has not yet found definitive evidence of psychological properties such as thought and intent and that it is an open question whether such properties may ever emerge. However, commercial LGAIMs are capable of advanced linguistic tasks such as holding a conversation, summarising text, and extracting information from data. Interfaces to LGAIMs have been built in the form of chatbots that allow anyone, including children, to interact with AI. Some of the ramifications are not unique to generative AI and may be gauged based on what we are learning for example, about the effects of substitution of offline activities and social interactions by online ones, about the impact of disinformation, or about the effectiveness of chatbot interventions. Other ramifications might be unique, however. From as soon as they have learned to talk, children can engage with artificial conversational agents, such as those that are currently on the market as smart speakers and trust these relatively more than humans for factual information (Xu, 2023). With generative AI, the range of topics for conversation becomes as broad as the internet. Furthermore, the sophisticated and adaptive conversational skills of applications that use LLMs may reduce the advantage that human social partners still enjoy on the trustworthiness of personal information.

### Generative AI in the real world

Having taught machines to generate language indistinguishable from our own is a profound moment in human history. While the evidence is inconclusive, with indications that overfitting may be responsible for some of the remarkable abilities reported as emerging from LGAIMs, the real-world implication remain historic. In 2022, one of Google’s engineers tasked with testing their LaMDA large language models was fired after claiming it to be sentient. What is important here is not whether the model was conscious or not, but that a person was willing to give up their job in defence of his beliefs about the model. So much so, people went as far as to question his mental health. This is likely the first of many such stories to come.

## Conclusions

Understanding the impact of the rapidly digitalising ecology of human development and testing ways to leverage technological advances to support families and young people are increasingly important themes in our field and journal. The current issue, for example, features the trial by Werner-Seidler and colleagues, showing how digital technology may not only disrupt sleep but can also be used to reduce insomnia. It may be a little too early to also turn to generative AI as a source of novel models of human mental health and functioning. However, people use language to create myth and legend, to create art and science, to create friendships and loving relationships. Now that LGAIMs can effortlessly produce such language if prompted well, new opportunities for redressing the social order may present themselves, for better or worse.
